# Direct Measurements of Turbulence in the Upper Western Pacific North Equatorial Current over a 25-h Period

**DOI:** 10.3390/s22031167

**Published:** 2022-02-03

**Authors:** Wenlong Yang, Hui Zhou, Yonggang Wang, Juan Liu, Hengchang Liu, Chenglong Liu, William Dewar

**Affiliations:** 1 Key Laboratory of Ocean Circulation and Waves, Institute of Oceanology, Chinese Academy of Sciences, Pilot National Laboratory for Marine Science and Technology, Qingdao 266237, China; yangwenlong16@mails.ucas.ac.cn (W.Y.); clliu@qnlm.ac (C.L.); 2 University of Chinese Academy of Sciences, Beijing 101408, China; 3 Key Laboratory of Marine Science and Numerical Modeling, Ministry of Natural Resources, First Institute of Oceanography, Qingdao 266061, China; ygwang@fio.org.cn; 4 Laboratory for Regional Oceanography and Numerical Modeling, Pilot National Laboratory for Marine Science and Technology, Qingdao 266237, China; 5 Shandong Key Laboratory of Marine Science and Numerical Modeling, Qingdao 266061, China; 6 Beijing Institute of Applied Meteorology, Beijing 100029, China; liujuan@mail.iap.ac.cn (J.L.); liuhengchang16@mails.ucas.ac.cn (H.L.); 7 State Key Laboratory of Geo-Information Engineering, Xi’an 710054, China; 8 Department of Ocean, Atmosphere and Earth Sciences, The Florida State University, Tallahassee 32304, FL, USA; wdewar@fsu.edu

**Keywords:** turbulent mixing, barrier layer, anticyclonic eddy, diurnal cycle, shear and stratification, double-diffusive

## Abstract

Measurements of the turbulent kinetic energy dissipation rate (*ε*) were conducted by a free-fall microstructure profiler in the western Pacific North Equatorial Current (WPNEC) during a continuous period of 25 h, from the sea surface to about 160 m depth. In the mixed layer (ML), *ε* values were typically on the order of 10^−8^∼10^−7^ W kg^−1^, and an obvious diurnal cycle existed in the upper 40 m of the surface mixing layer. Below the ML, *ε* was reduced to 10^−9^∼10^−8^ W kg^−1^ with some patches of high *ε* reaching 10^−7.5^ W kg^−1^. The barrier layer was identified in the nighttime with a maximum thickness of 20 m, and it was eroded by the advection of freshwater within the lower part of the isothermal layers associated with an anticyclonic eddy in the afternoon. A simple scaling relevant to shear (*S*^2^) instability and stratification (*N*^2^) that can predict turbulent dissipation rates in the transition layer, between the well-mixed layer and the thermocline below, was obtained through the scaling $\varepsilon ∼{\left|S\right|}^{-0.40}{\left|N\right|}^{0.20}$. Besides turbulence, double-diffusive processes also contributed to the vertical mixing levels in the upper WPNEC.

## 1. Introduction

Turbulent mixing plays a key role in the upper layer of the ocean, where active momentum, heat, and gas exchanges between the ocean and the atmosphere exist. It regulates water properties and drives ocean circulations, which ultimately modulate the climate system through affecting the large-scale heat budget [1,2,3,4,5]. The turbulent exchange of momentum and scalars at both the air–sea interface and the ocean mixed layer (ML) base are crucial for climate forecasts [6,7]. Large eddy simulation includes the energy pathway associated with the Craik–Leibovich interaction between the Stokes drift and turbulence and can better represent the ML under complex effects of wind stress, buoyancy flux, and surface waves [8,9,10,11]. However, our knowledge of the essential physics that govern the turbulent mixing in the ML is still quite limited, which results in typical large errors in boundary-layer thickness simulation in current climate models [12,13]. Therefore, direct measurements of the upper-ocean layer turbulence are very valuable for a better parameterization of mixing and its simulation [14,15].

**Figure 1:**
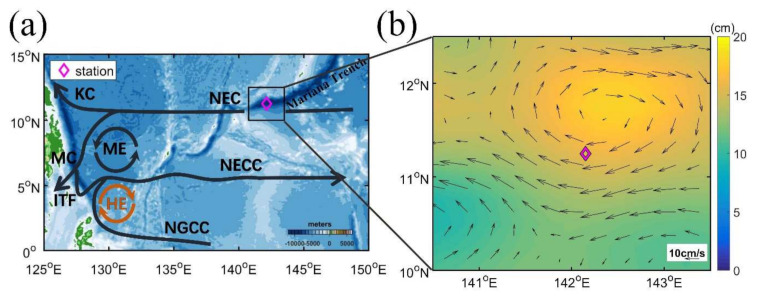
(**a**) Schematic of major surface currents in the tropical western Pacific Ocean. NEC, North Equatorial Current; KC, Kuroshio Current; MC, Mindanao Current; NECC, North Equatorial Counter Current; NGCC, New Guinea Coastal Current; ME, Mindanao Eddy; HE, Halmahera Eddy; ITF, Indonesian Throughflow. Color shading shows the topography from ETOPO2. (**b**) The surface geostrophic velocity anomalies (arrows) averaged from 8 to 9 April in 2018 from altimetry data with the synchronous sea surface height anomalies (SSHA) being superimposed. The purple diamond shows the microstructure profile measurement station (142.15° E, 11.25° N).

In the western Pacific Ocean (Figure 1a), the North Equatorial Current (NEC) flows westward between 8° and 18° N, and it splits into two western boundary currents near the Philippine coast [16,17], namely the northward Kuroshio Current (KC) and the southward Mindanao Current (MC). The high-salinity North Pacific Tropical Water (NPTW) around 24.0 $\sigma_{\theta }$ in the thermocline in the NEC enters into the tropical and subtropical gyre through the MC and KC [18,19], respectively, involving exchanges through mixing [20,21] and substantial water mass transformations, such as a decrease in salinity and an increase in characteristic density [22,23,24]. 

The NPTW can affect equatorial thermal structure by forming spiciness anomalies and barrier layers (BL), which can trap kinetic energy transferred from the wind to the ocean and modulate atmosphere–ocean interactions [25,26,27,28,29,30,31,32]. The BL is located between the bases of the ML and the isothermal layer (IL) and is characterized by strong salinity stratification and weak (or neutral) temperature stratification [25]. Interpreting the variability of the IL and ML in terms of surface-forced turbulent mixing can provide insight into the physical processes that are responsible for the formation of the BL in the NEC [33,34]. 

Given the dominance of evaporation over precipitation in the Pacific NEC in winter, the formation of the BL is suggested to be caused by the subduction of the NPTW [35]. Although the BL in this region was also identified in spring with a decreased thickness by previous studies [26,35], whether the subduction of NPTW results in its formation is not clear. Meanwhile, subsurface processes for the BL formation suggested by previous studies were based on heavily smoothed climatological fields of hydrographic data. Given that the establishment of a BL could be associated with small, transient, and irregularly distributed processes, understanding the detailed formation mechanism on the diurnal timescale is very important to understanding its climatology field. 

Previous studies suggest the important contribution of double-diffusive mixing processes to vertical mixing in the tropical western Pacific Ocean [36,37]. Lee et al. (2014) [38] first quantified the estimated flux and associated vertical diffusivity due to double diffusion in the western equatorial Pacific by using oceanic microstructure measurements in the upper 300 m, which are approximately one order of magnitude higher for temperature and density and two orders of magnitude higher for salinity compared to values calculated from a turbulence model. Despite the importance of double diffusion to vertical mixing and in regional water mass transformations [39,40,41], few quantitative works have examined the degree to which double-diffusive mixing may contribute to turbulent and vertical mixing levels in the NEC. 

Understanding surface turbulent mixing is crucial to investigating the dynamics and thermodynamics of the NEC and its contribution of mass, heat, and salt transport to the tropical-subtropical gyres [27,42]. Specifically, the diurnal variability associated with the wind, waves, and surface buoyancy fluxes plays an important role in the NEC’s upper ocean temperature and stratification, and potentially has a nonlinear rectified effect on longer time scales. Previous studies have revealed the equatorial ML as a region of intense and deep turbulent mixing with a large diurnal variability of turbulence [43,44]. However, our knowledge of the diurnal variation of turbulence in the western Pacific NEC remains quite limited, let alone during longer periods, including its influences on global climate change, which obscures our understanding of its role in the water mass transformation and air–sea interaction in the western Pacific Ocean. 

In this study, we analyze a 25-h long set of fixed-point direct microstructure profile (MSP) observations in the upper 160 m of the main axis of the western Pacific NEC (Figure 1). We focus on the diurnal changes in the surface forcing conditions and their effects on turbulence within the upper ocean from just below the ML up to the sea surface. Particular attention is paid to diurnal turbulent mixing associated with surface buoyancy fluxes, shear instability, and double diffusion in the surface mixing layer, the ML, and the transition layer (TL) in the NEC. 

The paper is organized as follows. Data and methodology are described in Section 2. The meteorological and oceanic conditions during the data collection are presented in Section 3. In Section 4, the characteristics and dynamics of diurnal turbulent mixing in the WPNEC are presented, and the discussion and conclusions are given in Section 5.

## 2. Observations and Methods

Observations were obtained around the Mariana Trench in the western Pacific NEC main axis from 8 to 9 April 2018 by R/V *XIANGYANGHONG18* of the First Institute of Oceanography, Ministry of Natural Resources of China (Figure 1). Turbulence measurements were taken using a free-fall microstructure profiler (MSP) MSS60 manufactured by Sea & Sun Technology, equipped with two fast response shear probes, one fast and one ordinary response temperature probe, and one ordinary conductivity cell. The sinking velocity of the profiler was over 0.7 m s^−1^ during the 25-h observation (Supplementary Figure S1), which satisfies the requirements of time response of the shear probe. In total, 53 MSP casts were conducted with a sampling frequency of 1024 Hz from the sea surface down to depths of 130–160 m, depending on weather and oceanographic conditions, at the station (142.15° E, 11.25° N) from 2145 local time (LT) on 8 April to 2245 LT on 9 April in 2018. Seven CTD casts were conducted with one cast from the surface to 5622 m, and the others were down to about 250 m. The shipboard meteorological measurements of wind speed and direction, air temperature, and atmospheric pressure acquired every 10 s were averaged into the 10 min resolution for this study.

Current velocity was collected using a Teledyne Marine WorkHorse Sentinel 300 kHz lowered Acoustic Doppler Current Profiler (LADCP) attached to a CTD frame about a few minutes before each MSP measurement with the maximum depth ranges between 160 m and 300 m. The LADCP was configured to ping about once per second with the first ping length of 6.24 m, and velocities were estimated in 4-m cells. We used the Lamont-Doherty Earth Observatory (LDEO) software to process the LADCPs data, which is based on an implementation of the velocity inversion method [45,46]. The advantage of this method is that the uncertainty can be estimated in deriving the absolute velocity [45]. Unfortunately, the bottom-tracking data were not available during the observation. We used the GPS data to remove the ship drift effect on the LADCP measurements. Although the derived absolute velocity may be affected by the lack of bottom-tracking, the velocity shear is not affected. The shear variance (*S*^2^) was calculated based on the LADCP measured zonal (u) and meridional (v) velocities, where *S*^2^ = (du/dz)^2^ + (dv/dz)^2^.

The turbulent kinetic energy (TKE) dissipation rate (*ε*) was calculated based on the method proposed by Roget et al. (2006) [47], which fits the Nasmyth spectrum to the directly measured small-scale shear power spectra over segments of 2 s (corresponding to a vertical spacing of ∼1.2 m), and the wavelet procedure was applied to small-scale shear signals before calculating the *ε* to remove noise. The diapycnal diffusivity *𝜅_𝜌_* was estimated based on the formula proposed by Osborn (1980) [48], *𝜅_𝜌_* = 0.2 *ε*/*N^2^*, where 0.2 is the assumed constant mixing efficiency and *N^2^* is the squared buoyancy frequency. Due to contamination by the ship’s wake, the turbulence measurements and thus the estimated *ε* and *𝜅_𝜌_* were unreliable in the top ∼10 m, these data were therefore excluded from further analysis.

Daily gridded maps of merged absolute dynamic topography heights (MADT-H) and absolute geostrophic velocities (MADT-UV) from Aviso+ (http://www.aviso.altimetry.fr, accessed on 15 April 2019) and the Copernicus Marine Environment Monitoring Service (CMEMS http://marine.copernicus.eu/, accessed on 15 April 2019) were used to infer the surface currents during the period of the MSP observations. Longwave radiation and shortwave radiation data computed by the European Centre for Medium-Range Weather Forecasts (ECMWF) forecast system with a resolution of 0.125° × 0.125° were used in the buoyancy flux calculation [49] (http://apps.ecmwf.int/datasets/, accessed on 15 April 2019).

Surface buoyancy flux $J_{b}^{0}$ was calculated following formulas given by Shay and Gregg (1986) [50], and it contains contributions from the surface heat flux, $J_{q}^{0}$, and the surface salt flux, $J_{s}^{0}$:

(1) $$J_{b}^{0}=\frac{-g}{\rho_{w}}\left[\frac{\alpha }{c_{p}}J_{q}^{0}+\beta J_{s}^{0}\right]\;=\frac{g}{\rho_{w}}\left[\frac{-\alpha }{c_{p}}J_{q}^{0}+\frac{\beta s}{L_{E}\left(1-s\right)}J_{q}^{e}\right]$$

(2) $$J_{q}^{0}=J_{q}^{sw}+J_{q}^{lw}+J_{q}^{e}+J_{q}^{s}$$

where *g* is gravitational acceleration (9.8 m s^−2^), $\rho_{w}$ is the density of seawater (1025 kg m^−3^), $c_{p}$ is the specific heat of seawater at constant pressure (4.1 × 10^3^ J K^−1^ kg^−1^), α is the coefficient of thermal expansion (−2 × 10^−4^ K^−1^), β is the coefficient of haline contraction (0.76 PSU^−1^ = 0.76 × 10^−3^ kg g^−1^), and $L_{E}=2.5\times 10^{6}\left({J\;\mathrm{kg}}^{-1}\right)$ is the latent heat of evaporative heat flux. $J_{q}^{sw}$ and $J_{q}^{lw}$ are the shortwave radiative flux and longwave radiative flux, respectively, which are provided by the European Centre for Medium-Range Weather Forecasts. $J_{q}^{s}$ and $J_{q}^{e}$ are the sensible heat flux and the latent heat flux, respectively, which are calculated using the bulk aerodynamic formula by Fairall et al. (1996) [51] from the data of the automatic weather station on the R/V Xiangyanghong 18. 

We also calculated the Monin–Obukhov length, $L=-u_{*}^{3}/kJ_{b}^{0}$ (where $u_{*}=\tau /\rho_{w}$ is the friction velocity and k = 0.41 is von Karman’s constant), to estimate the relative strength of the wind stress-induced mechanical TKE production term and buoyancy production term within the ML. The positive L represents buoyantly stable conditions and negative L represents an unstable buoyancy profile.

The layer between the bases of the ML and the isothermal layer has been referred to as the BL [25] and is normally characterized by strong salinity stratification and weak (or neutral) temperature stratification. Here, we used the gradient criterion to determine the isothermal layer depth (ILD) and the mixed layer depth (MLD) given that the evident difference between the isothermal (isopycnal) layer and thermocline (pycnocline) is the vertical gradient [52]. We followed the definition of Chu et al. (2002) [53] to define the ILD as the depth where the vertical temperature gradient was larger than 0.05 °C m^−1^, and the MLD was calculated as the depth where the vertical density gradient was larger than 0.015 kg m^−4^ following [25]. The ILD and MLD were almost the same if we use the criteria of 0.02 °C m^−1^ and 0.015 kg m^−4^, respectively. Here, we chose the threshold of 3-dbar for the BL thickness, given the errors in computing, and ILD and MLD were 1-dbar and 2-dbar for the CTD measurement accuracy of 0.02 °C, respectively. The interface between the stratified, weakly turbulent interior and the strongly turbulent surface mixed layer is referred to as the transition layer [33,54], which is defined as the depth of maximum stratification N^2^.

The density ratio was used to classify the double diffusion contribution to the vertical mixing, *R_ρ_* = *α*$\frac{\partial \theta }{\partial z}$/*β*$\frac{\partial S}{\partial z}$ [55], where *α* = −$\rho_{w}$^−1^ is the thermal expansion coefficient and $\rho_{w}$ is the density of sea water, *β* = −$\rho_{w}$^−1^$\frac{\partial \rho_{w}}{\partial S}$ is the haline contraction coefficient, the vertical coordinate is taken to be positive upward. Here, to avoid extremely large *R_ρ_* values that alternate in sign, we used the Turner angle, $T_{u}=\arctan((\alpha \bar{\frac{\partial \theta }{\partial z}}-\beta \bar{\frac{\partial S}{\partial z})}/(\alpha \bar{\frac{\partial \theta }{\partial z}}+\beta \bar{\frac{\partial S}{\partial z}}))$, to discuss different types of double diffusion [56,57]. *R_ρ_* and $T_{u}$ are related by *R_ρ_* = −tan($T_{u}$ + 45°). The water column was doubly stable for −45° < Tu < 45° (or 0 < *R_ρ_* < ±∞), “diffusive” double diffusion when −90°< Tu < −45° (or 0 < *R_ρ_* < 1), and “salt-fingering” double diffusion when 45° < Tu < 90° (or 1 < *R_ρ_* < ±∞). 

The buoyancy Reynolds number ($R_{eb}$) was calculated according to Gregg (1988) [58] and Inoue et al. (2007) [59]: $R_{eb}=\varepsilon /\left(\nu N^{2}\right)$, where ν is the kinematic viscosity, and $\nu =1.0\times 10^{-6}m^{2}/s$. The temperature ($K_{T}^{f})$ and salinity ($K_{S}^{f})$ diffusivity caused by salt-fingering was calculated according to Schmitt (1981) [60] and Merryfield et al. (1999) [61]:

(3) $$K_{S}^{f}=\frac{K^{*}}{1+{\left(R_{\rho }/R_{c}\right)}^{n}},\;R_{\rho }>1\;\;and\;\;\partial_{z}\rho <0$$

$K_{S}^{f}=0$ otherwise;

(4) $$K_{T}^{f}=0.7K_{S}^{f}/R_{\rho }\;$$

where *R_c_* = 1.6 and *n* = 6. $K^{*}$ is set to be $1\times 10^{-3}m^{-2}s^{-1}$ to yield diffusivities comparable to those inferred from microstructure measurements.

A conceptual schematic of the upper ocean boundary layer in Callaghan et al. 2014 [62] (their Figure 1) will help to understand related concepts mentioned in this section.

## 3. Meteorological and Hydrological Conditions

Since an understanding of the mean and fluctuations of the meteorological and hydrographic conditions is necessary to interpret the microstructure data, we first present the atmospheric conditions during the MSP observations, followed by a discussion of the observed turbulent variables. 

**Figure 2:**
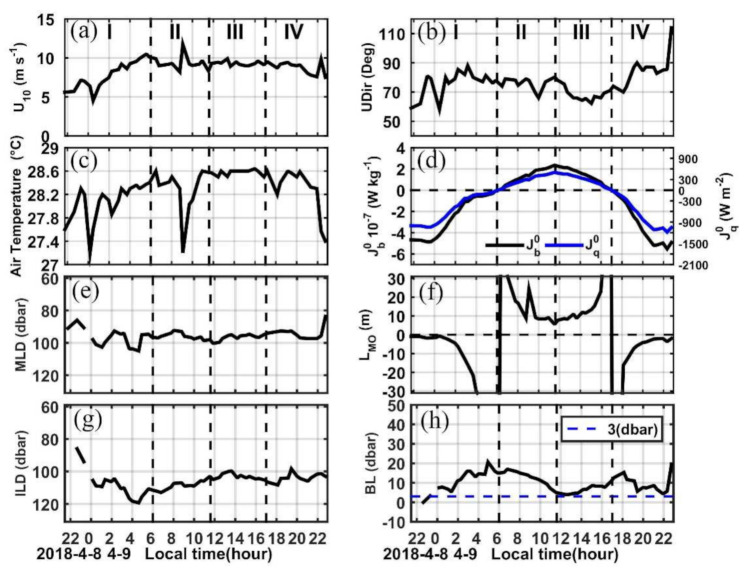
Time series of (**a**) wind speed (U_10_), (**b**) wind direction (U_dir_, 0° implies wind from the north), (**c**) air temperature (°C), (**d**) surface buoyancy flux ($J_{b}^{0}$), and surface heat flux ($J_{q}^{0}$), both are positive when the ocean gains heat from the atmosphere. (**e**) Mixed layer depth (MLD), (**f**) Monin–Obukhov length (L), (**g**) isothermal layer depth (ILD), and (**h**) barrier layer thickness (BL). The four time periods are labelled in panel (**a**) and indicated in each panel thereafter by vertical dashed black lines. To eliminate very sharp spikes, a 3-point running mean is adopted for the MLD, ILD, and BL, respectively.

We divided the dataset into four time periods based on the surface buoyancy flux (Figure 2d). Period I is characterized by negative heat flux (heat loss from the ocean) and extends from the beginning of the measurement to 0600 LT. Period II is from 0600 LT to 1200 LT, when the heat flux shifted to positive and reached its maximum. Period III is from 1200 LT to 1700 LT, when the positive heat flux decreased and reached zero. Period IV is from 1700 LT to the end of the measurements when the heat flux shifted to negative. Table 1 provides the average values of the wind speed (U_10_), surface buoyancy flux ($J_{b}^{0}$), Monin–Obukhov length (M_o_), MLD, and BL thickness for each time period along with the number of MSP casts conducted (N). We will discuss these four periods in more detail in the following sections.

Figure 2 shows the time evolution of several meteorological and oceanic variables during the 25-h MSP measurement from 8 to 9 April 2018. Wind speeds were moderate with an averaged value of ∼8.6 m s^−1^, and two wind gusts up to 12 m s^−1^ occurred at 0900 LT and 2230 LT, respectively. These were associated with a drop in air temperature up to 1.4 °C (Figure 2c). As we will see in Section 4.2, these wind gusts strongly affected the diurnal thermal cycle. The wind direction was mostly northeasterly indicating the typical trade winds there, and it became more easterly towards the end of the observation period (Figure 2b). Although no wave observations were made, the visual inspection suggested that the sea state was relatively calm, and no rainfall occurred during the 25-h observation.

## 4. Results

### 4.1. The Barrier Layer

**Figure 3:**
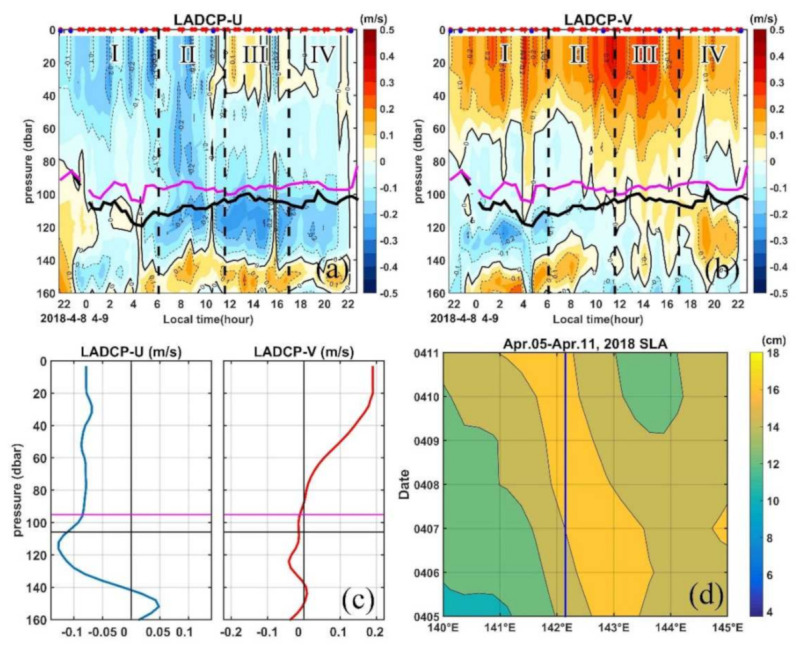
Time series of current profile measured by LADCP for (**a**) zonal, (**b**) meridional, (**c**) mean profile of the horizontal velocities and (**d**) time-longitude plot of the sea level anomalies at 11.25° from altimetry data from 05 to 11 April 2018. Purple and black lines in (**a**–**c**) mark the MLD and ILD, respectively, and the blue line in (**d**) indicates the longitude (142.15° E) of the MSP station during the 25-h observation period. Red and blue dots in the top axis of (**a**,**b**) indicate the MSP/LADCP and CTD cast, respectively.

The geostrophic velocity anomalies averaged between 8 April (Day 1) and 9 April (Day 2) 2018 based on altimetry data clearly show that the MSP station is located in the south part of an anticyclonic eddy (AE) centered around (11.7° N, 142.2° E) with a diameter of 200 km (Figure 1b). The time series of the current profile measured by LADCP show that the current is northwestward in the upper 80 m during our observations; however, a northeastward flow occurs in period III (Figure 3a,b). The mean velocity profiles show that the westward velocity is dominant in the upper 120 m ranging between 0.06 m s^−1^ and 0.13 m s^−1^, and the northward velocity can reach 0.19 m s^−1^ in the upper 20 m and decreases sharply and reverses its direction around 70 m (Figure 3c). There is a strengthening of the meridional velocity from period I to period III, which may be caused by the advection associated with the westward propagation of the AE as indicated from the synchronous anomalies of sea level and geostrophic velocity from altimetry data (Figure 3d and Figure S2). The AE passes by the MSP station during the 25-h measurements with a mean westward propagation speed of ∼0.2 m s^−1^ (Figure 3d), which is equivalent to about 18 km displacement during the 25-h observation. This suggests that the lateral advection associated with the AE may influence the dynamics of the diurnal cycle of the turbulence during our 25-h MSP observation. 

**Figure 4:**
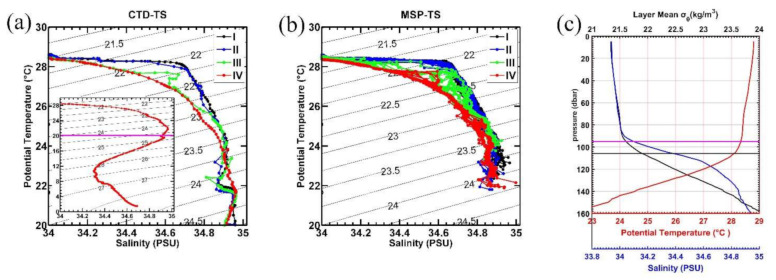
Potential temperature salinity diagram observed from (**a**) CTD and (**b**) MSP. The inserted T-S diagram in (**a**) is from the full-depth CTD cast with the purple line indicating the 20 °C. (**c**) Vertical profile of the mean potential temperature (°C; red), salinity (psu; blue), and density (kg m^−3^; black) during the MSP observation. The purple and black lines are the mixed layer depth and isothermal layer depth.

We first present the potential temperature–salinity (*θ*-*S*) relationship given its important role in controlling the water density and governing the vertical movement of ocean waters. Figure 4a,b shows the *θ*-*S* diagram observed by the CTD and MSP at the station, respectively. The inserted *θ*-*S* curve from the deep CTD cast in Figure 4a shows the typical mirrored S-shaped character of northwestern Pacific Ocean water masses [22]. Both the CTD and MSP measurements show the tropical surface water (TSW) with 26° < *θ* < 30 °C and 33.5 < *S* < 34.6 psu above 23 σ_*θ*_, which is formed locally in the vicinity of the intertropical convergence zone (ITCZ; [22]). Below the 23.5 σ_*θ*_ surface, high salinity water (34.8 < *S* < 35.0 psu) can be seen, which is the NPTW, forming around 20° N, 140° E–160° W due to excessive evaporation there [20,63]. Generally, the CTD and MSP show consistent features of the water property, suggesting the reliability of the observations. 

Variation in the *θ*-*S* relationship at a fixed density is often referred to as spiciness [64,65], because water on an isopycnal can be either relatively cold and fresh, or hot and salty. We can see obvious spiciness in the *θ*-*S* diagram from periods I to IV with the *θ*-*S* curve being pulled toward colder and fresher values on isopycnals above 23.5 σ_*θ*_ surface (Figure 4a,b). The monotonic change of water masses with time suggests the advection effects associated with the AE on the water mass evolution, which can also be supported by distinct water properties between periods I-II and periods III-IV (Figure 4a,b). 

The mean vertical profiles of the temperature and salinity observed by the MSP are shown in Figure 4c. The temperature profile shows a vertically uniform distribution of 28–29 °C in the top 100 m with a sharp decrease to 22–23 °C around 160 m, and the isohaline layer of 34.0 psu is about 10 m shallower than that of the temperature with a rapid increase to 34.95 psu around 160 m. The shallower depth of the isohaline layer than that of the isothermal layer suggests the existence of a BL, where strong salinity stratification and weak temperature stratification exist. Using the gradient criterion, we calculate the mean ILD and the MLD during the MSP observation period in Figure 4c, which clearly shows the existence of BL with a mean thickness of 11 m. This is comparable to the spring climatological values of the BL in this region given by Sprintall and Tomczak (1992) [26].

**Figure 5:**
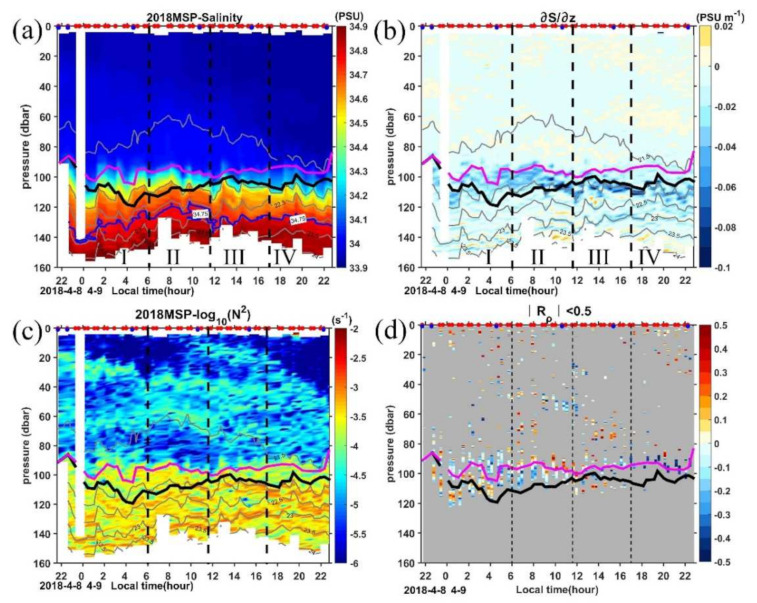
Time series of (**a**) salinity (S), (**b**) vertical salinity gradient (∂S/∂z, negative values indicate S increase with depth), (**c**) the buoyancy frequency (N^2^), and (**d**) the density ratio (*R_ρ_*) profile with $\left|R_{\rho }\right|\;<0.5$ being color shaded and gray otherwise. In each panel, dashed black lines mark the four periods, gray solid lines are the isopycnals, thick purple and black lines mark the MLD and ILD, respectively. Red and blue dots in the top axis indicate the MSP/LADCP and CTD cast, respectively.

Significant variations can be seen in the thickness of the BL ranging from 0 m to 20 m during the 25-h observation with two peaks occurring around LT0500 and LT1700, respectively (Figure 2h). The first peak is generally consistent with the time when the diurnal thermocline, which is formed at a certain depth during the day in the ML while a temperature gradient remains small near the surface, reaches its maximum depth at the transition between nighttime cooling and daytime heating, and the surface heat flux reverses sign (Figure 2d). The wind increases from 2 m s^−1^ at 0030 LT to 10 m s^−1^ at 0500 LT (Figure 2a), and the ocean is losing heat before 0600 LT (Figure 2d); therefore, both heat loss and strong wind force favor the occurrence of a stratification-formed BL. The sharp vertical salinity gradient (>0.03 psu m^−1^) associated with the salinity front between the TSW and NPTW in the BL during period I-II also supports the mechanism (Figure 5a,b). 

The above BL is calculated based on the gradient criterion, which is a common approach using two independent definitions for the halocline and the thermocline, respectively. However, this approach cannot capture the relative contributions to the stratification of the water column by the salinity and temperature, respectively [66], so we further calculate the density ratio, *R_ρ_*, to classify the contribution by salinity in the BL. If *R_ρ_* < 0, the water column is stable, and there are no overturns, otherwise, overturns associated with double diffusion or salt-fingering may happen [56]. The large contribution of salinity to the stratification in the BL indicates the value of *R_ρ_* being equal to or very close to zero. 

Looking at the vertical profile of N^2^ and *R_ρ_*_,_ we choose a threshold of *R_ρ_* = 0.5 to derive the extent of the BL (Figure 5c,d). During periods I-II, bands of *R_ρ_* < 0.5 in the depth range 80–120 m are generally consistent with the BL calculated based on the gradient criterion, albeit the double thickness, suggesting the dominant contribution of salinity to the density in the BL. However, during periods III-IV, there are almost no values of *R_ρ_* < 0.5 in the BL, only a few *R_ρ_* < 0.5 appear above the ML, suggesting the salinity effect in the BL is quite weak. The profile of the salinity vertical gradient also confirms the existence of a BL with an obvious halocline during periods I and II, and relatively uniform salinity stratification in BL during periods III and IV (Figure 5b). This suggests that the gradient criterion cannot capture the real BL in the latter two periods. The erosion of halocline during periods III-IV seems to be caused by the significant decrease in salinity in the ML (Figure S3), which can be seen from the downward slant of the isopycnals beginning around 0900LT (Figure 5a). As there was no rain during the observation, the significant decrease of salinity is likely due to the advection associated with the AE (Figure 3d). The downwelling in the AE will deepen the fresh TSW and relatively deep ML, resulting in the erosion of the halocline by strong lateral advection of the TSW when passing by the MSP station. 

### 4.2. Turbulence Characteristics

#### 4.2.1. Evolution of *ε* in the Mixed Layer

**Figure 6:**
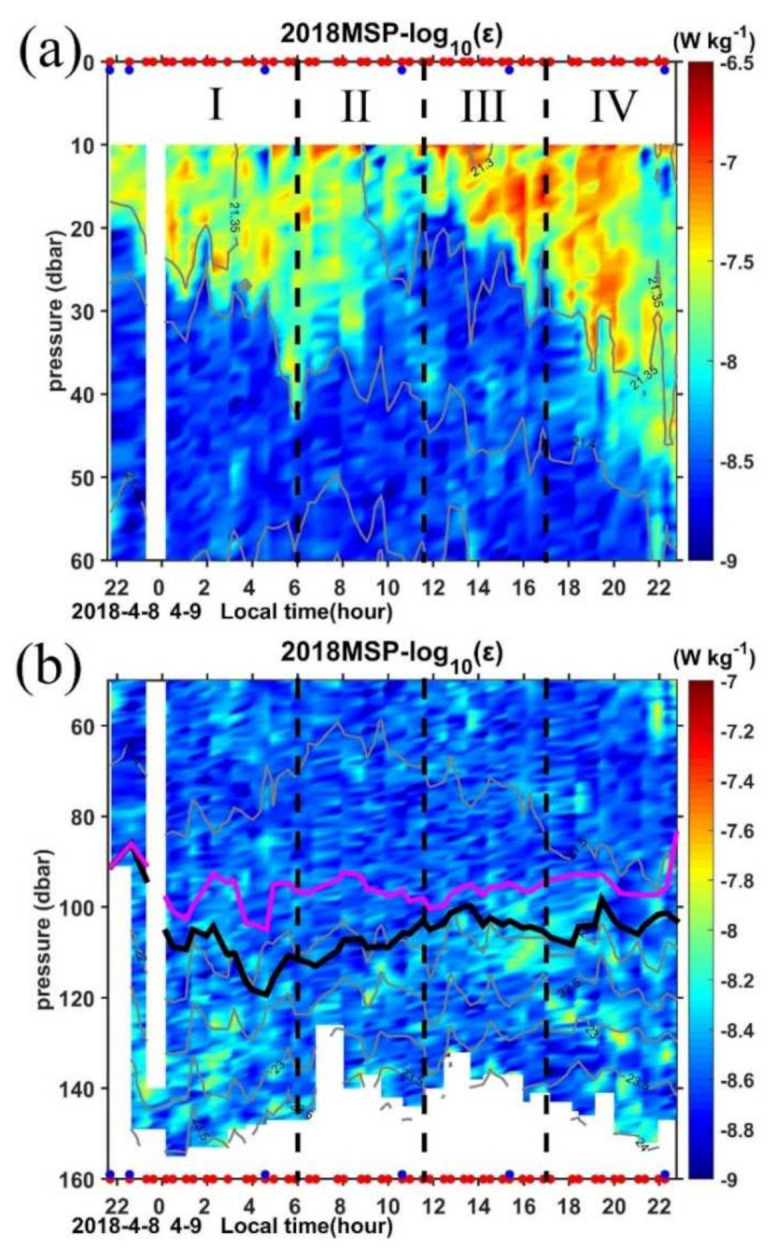
Time series of turbulence kinetic energy dissipation rate (*ε*) for (**a**) upper 60 dbar and (**b**) 60–160 dbar. Purple and black lines mark the MLD and ILD, respectively. Red and blue dots in the top and bottom axis of (**a**) and (**b**) indicate the MSP/LADCP and CTD cast, respectively.

The *ε* shows quite large fluctuations between the water column in the upper 40 m and that below (Figure 6). In the upper 40 m, the order of magnitude values of *ε* is typically 10^−8^∼10^−6.5^ W kg^−1^ with an obvious diurnal cycle, whereas it drops to 10^−9^∼10^−8^ W kg^−1^ below. These two distinct regions correspond to the mixing layer, where surface fluxes are being actively mixed by turbulent processes, and the ML, where surface fluxes have been mixed in the past one day or more [53,67,68], respectively. The surface mixing layer zone is also characterized by intermittent density inversions with a thickness of 20–25 m (Figure S4). 

During period I, the negative Monin–Obukhov length (−33.19 m) suggests convective conditions, so the depth of relatively uniform values of the *ε* between 10^−7.5^∼10^−6.5^ W kg^−1^ show a deepening tendency caused by enhanced driven mixing under nighttime convection with the maximum depth of 40 m occurring at 0600 LT. Although heat fluxes in period II and period III are both positive (Figure 2d), the dissipation rates in the mixing layer are quite different from each other. During period II, as the heat fluxes change from heat loss to heat gain, the *ε* drops to 10^−7.5^∼10^−8^ W kg^−1^, which is consistent with the reduced turbulence production due to stable stratification caused by the input of solar radiation during the daytime [44].

**Figure 7:**
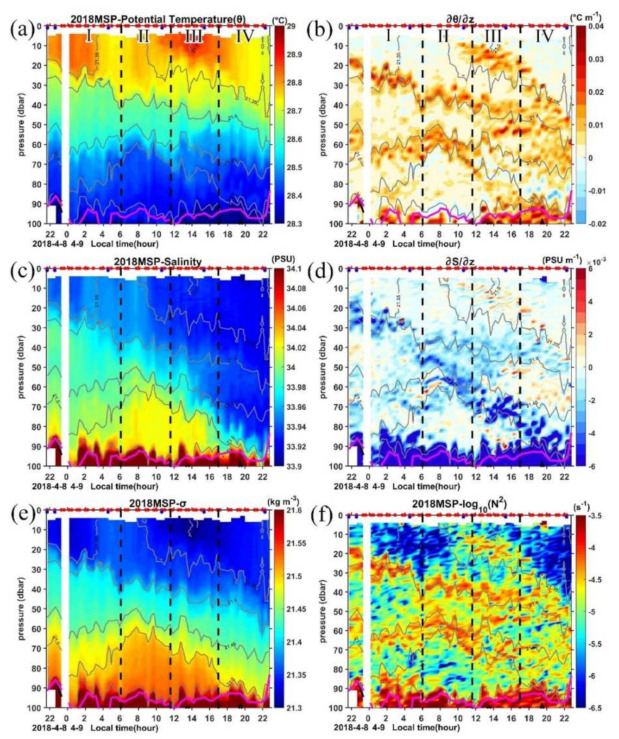
Times series of (**a**) potential temperature (θ), (**b**) vertical temperature gradient (∂θ/∂z, negative values indicate θ increase with depth), (**c**) salinity (S), (**d**) vertical salinity gradient (∂S/∂z, negative values indicate S increase with depth), (**e**) specific density (σ), and (**f**) the buoyancy frequency (N^2^) for the upper 100 m. In each panel, dashed black lines mark the four periods, gray solid lines are the isopycnals, thick purple line marks the MLD. Red and blue dots in the top axis indicate the MSP/LADCP and CTD cast, respectively.

**Figure 8:**
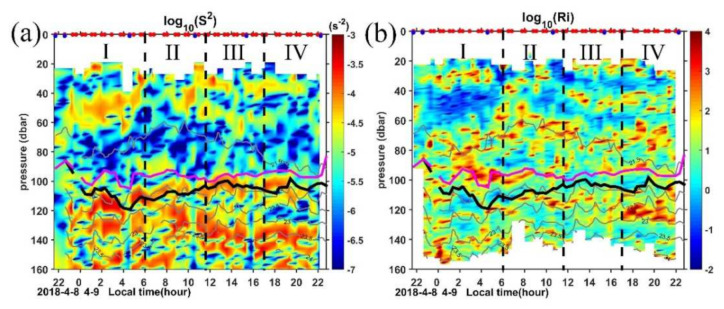
(**a**) Vertical shear squared (*S^2^*) and (**b**) corresponding gradient Richardson number *R_i_* calculated from the LADCP measurements. Gray lines are the isopycnals. Purple and black lines mark the MLD and ILD, respectively. Red and blue dots in the top axis indicate the MSP/LADCP and CTD cast, respectively.

However, high *ε* (on the order of 10^−6.5^ W kg^−1^) occurs in almost the full water column in the upper 20 m layer in period III (Figure 6a). Previous studies suggest that the surface mixing layer could trap the wind momentum in this isolated layer and form a diurnal jet due to the weak Coriolis turning in the top 10–15 m in the tropics [33,69]. To further explore if this is the mechanism for the occurrence of high *ε* during period III in the mixing layer, we check the evolution of the zonal and meridional velocities in the top 20 m obtained by the LADCP during the 25-h MSP observations (Figure 3a,b). The northwestward current turned to northeastward during period III with an increased amplitude, which indicates a diurnal jet with an increase in surface current along the persistently blowing northeasterly wind direction during this period. Warm water is trapped in this layer with the temperature being over 28.85 °C (Figure 7a), and a very sharp increase of the buoyancy frequency occurs at the base of this layer (Figure 7b–f), which prevents the water below it from the influence of surface forcing. The strong shear between 20 and 40 m from 1000 LT to 1400 LT also suggests the existence of the diurnal jet (Figure 8a). Below this high *ε* region, the dissipation decays sharply to 10^−8^ W kg^−1^ and is termed as the remnant layer, within which the energetics balance is between the change rate of TKE and *ε* [70]. 

In period IV, as the buoyancy and heat fluxes return to conditions of ocean heat loss, the convection is enhanced and the mixing is strengthened, the high *ε* region extends deeply and reaches 50 m at 2200 LT. Compared with period I, although both periods are nighttime convective conditions, the *ε* in period IV is much higher than that in period I. The relatively strong wind forcing in this period may contribute to the high *ε* by providing mixing in addition to the conversion of potential energy to kinetic energy (Figure 6a). Meanwhile, the different waters carried by the AE may also contribute to the enhanced mixing through double-diffusive mixing, which will be discussed in Section 4.3.

#### 4.2.2. Evolution of *ε* below the Mixed Layer

Compared with dissipation in the mixed layer, *ε* below 90 m is greatly reduced, consistent with the strong stratification in the pycnocline (Figure 6b). The order of magnitude values of background *ε* in this region are typically 10^−9^∼10^−8^ W kg^−1^ with some patches of high values reaching 10^−8^∼10^−7^ W kg^−1^. Previous study suggested that this regime also contained the entrainment zone, where turbulence is generated by shear, Kelvin–Helmholtz instabilities, internal waves, and overshooting thermals [71]. In general, the dissipation in the pycnocline does not follow a diurnal cycle, but if we compare the *ε* among the four periods, we can find that more high *ε* patches occur in periods I, III, and IV. During period I and IV, while the ocean is experiencing strong winds and convective cooling (Figure 2a,d), hot spots of strong *ε* may occur in a strongly stratified layer where shear drives the Richardson number (*R_i_*) to the critical value for turbulence (Figure 8b).

Period III is characterized by oceanic buoyancy gain, and convection is greatly reduced. However, some patches of high *ε* (∼10^−7.5^ W kg^−1^) occur just in and below the reforming BL in this period. Usually, the BL forms a barrier to the entrainment and turbulent mixing of cold thermocline water into the mixed layer and inhibits the downward momentum transport [28]. The high dissipation patches in this period also suggest the weak halocline in the BL. The high dissipation may be induced by the strong shear associated with the influence of the AE (Figure 8, Figure 3 and Figure S2). The strong shear resulting in a critical Richardson number where Kelvin–Helmholtz instabilities appear, induces turbulent mixing. We will discuss this shear induced instability in the following subsection.

#### 4.2.3. Shear and Stratification

**Figure 9:**
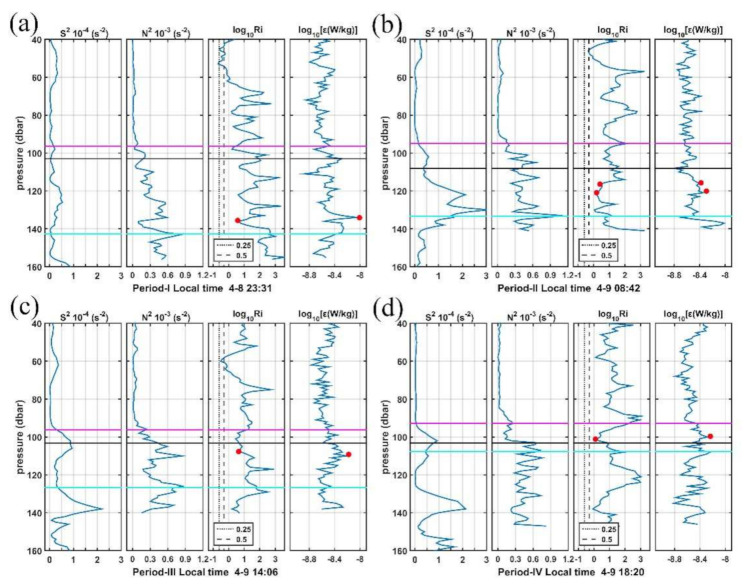
Vertical shear squared (*S^2^*), squared buoyancy frequency (*N^2^*), Richardson number (*R_i_*), and turbulence kinetic energy dissipation rate (*ε*) profiles for four casts (**a**–**d**) with LADCP measurements for each period. Black dashed line and dot-dashed line mark the value of *R_i_* = 0.25 and *R_i_* = 0.5, respectively. Red dots mark the high *ε* corresponding to the low *R_i_* in the transition layer. Purple, black, and cyan lines mark the MLD, ILD, and transition layer (TL), respectively.

The interaction between forcing and the static and dynamic states of the water, i.e., stratification and the shear, affect the response to forcing significantly. To explore the mechanism for these high *ε* patches, we present the evolution and vertical structures of shear, stratification, and turbulent mixing for those MSP casts with high *ε* patches (Figure 9). To reduce the noise, we average three consecutive MSP casts in each period for those high dissipation patches.

Although much variability occurs in the upper 160 m profiles taken during the 25-h observation, three distinct regimes appear in all profiles for the four periods. The surface zone, where the mixing layer is located, is characterized by a high dissipation rate (*ε* ≥ 10^−6.5^ W kg^−1^) and intermittent density inversions with a thickness of 20–25 m (Figure S4). Below is the well-mixed central zone extending to the base of the mixed layer around 80–100 m: it is marked by very uniform *ε* with the mean value near 10^−9^∼10^−8^ W kg^−1^. Below the base of the ML, a zone ranging between 100 and 140 m containing the IL and BL, exhibits both strong shear and stratification. This region is called the transition layer (TL), where exchanges of energy and shear between the mixed layer and the upper thermocline take place. The depth of TL fluctuates from 145 m during the 2331 LT cast in period I to 105 m during the 1820 LT cast in period IV. Currently, our understanding of the TL is very limited, and its effects are parameterized in general circulation models. Our observations suggest that the *ε* generally peaks at those depths where strong shear has occurred (*Ri* close to 0.5 or 0.25; red dots in Figure 9) in the TL. The situations where the depths between the low *Ri* and *ε* peaks differ may be attributed to both the coarse vertical resolution of the LADCP measurements (4 m cell) and the time lag between the MSP and LADCP casts (usually MSP was conducted a few minutes after the LADCP). Zhang et al. (2018) [72] also suggest the relatively strong parameterized mixing rate associated with fine-scale turbulent shear between 100 and 300 m at 12–14° N along the 142° E section based on analyses of ADCP measurements.

Conventional wisdom suggests that mixing rates transit from high values in the ML to low values in the TL, so the wind-driven momentum and shear can penetrate deeper into the interior [54]. However, during our observations, much higher dissipation rates caused by local shear instabilities occur in the TL than those in the ML, suggesting the influence of the TL on the ML. The observed shear instability at the base of the ML supports the typical processing of the diapycnal mixing parameterization in general circulation models (GCMs), which is represented by switching on extra mixing when the local *R_i_* drops below a critical value [6,33,73].

**Figure 10:**
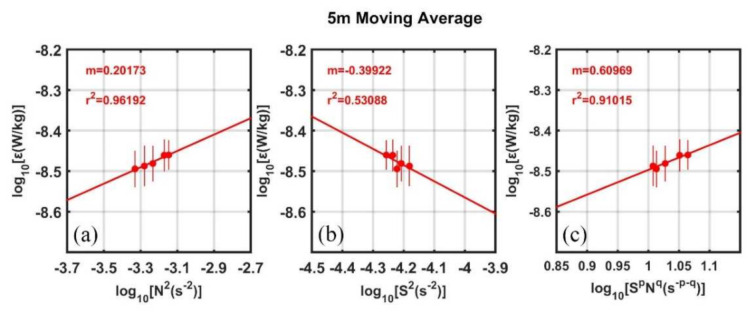
5 m Moving Average of scalings of turbulent dissipation rate *ε* in the TL with (**a**) N^2^, (**b**) *S*^2^, and (**c**) $\varepsilon ∼{\left|S\right|}^{p}{\left|N\right|}^{q}$ for *p* = −0.40 and q = 0.20, *p* and q are the slopes of the respective fit to *S*^2^ and N^2^. Least squares fits are computed using bin- and survey-averaged data (red dots) in the TL. The slope of the fit ‘m’ and coefficient of determination ‘r^2^’ are indicated at the top left in each plot. Error bars indicate 95% confidence intervals.

Since there are strong vertical shear and stratifications in the TL with the respective maxima being slightly offset in depth [74,75], here we try to look for simple scalings relevant to shear instability and stratification that can predict turbulent dissipation rates in the TL using the observed fine-structure data during this cruise. Following Sun et al. (2013) [76], the log-log scalings of survey-averaged *ε* versus N^2^ and *S*^2^ are presented in Figure 10a,b, respectively. The correlation between *ε* and N^2^ is much higher than that between *ε* and *S*^2^ (r^2^ = 0.96 vs. 0.53), suggesting that N^2^ is a better predictor for *ε* compared with *S*^2^. Moreover, the best-fit slopes for *ε* versus N^2^ (m = 0.20) and *S*^2^ (m = −0.40) are similar in amplitude but in opposite signs. By combing N^2^ and *S*^2^, we can find the best scaling $\varepsilon ∼{\left|S\right|}^{-0.40}{\left|N\right|}^{0.20}$, which can predict variations in *ε* in the TL with the correlation coefficient r^2^ = 0.91.

**Figure 11:**
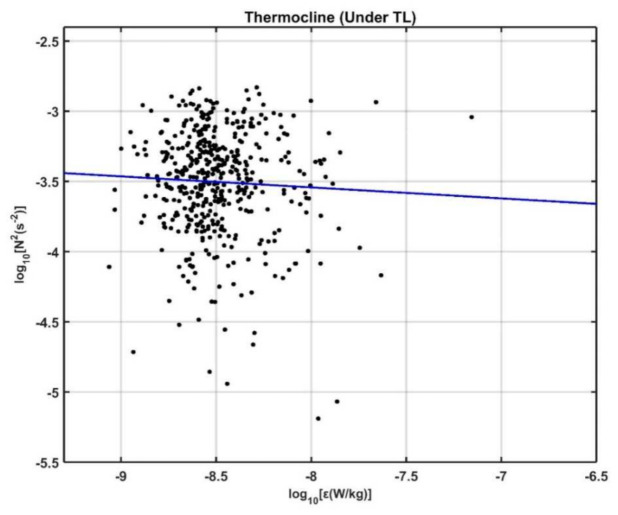
Scatter plot of N^2^ versus turbulent kinetic energy dissipation *ε* for observations in the thermocline (below the TL). The blue line indicates *ε* ∝ N^2^.

Previous studies suggested dissipation induced by internal wave–wave interactions scaled with N^2^, such that *ε* ∝ N^2^ [77,78], although Polzin et al. (1996) [79] argued that this scaling may not be appropriate for internal wave–flow interactions. Here, we check the validation of the above scaling between *ε* and the buoyancy frequency using the 25-h MSP measurements in the upper thermocline in the NEC (Figure 11). The scatter plot of N^2^ versus *ε* for our observations shows a large deviation from the *ε* ∝ N^2^ scaling with a trend of weak monotonic increase in dissipation with decreasing N^2^ (Figure 11). The inconsistency may suggest the influence of mesoscale flow associated with the AE (recall Figure 1b), as the interaction between shears induced by mesoscale flow and the internal wave field can cause enhanced mixing where the flow has a Richardson number <20 [79,80].

### 4.3. Double Diffusion

**Figure 12:**
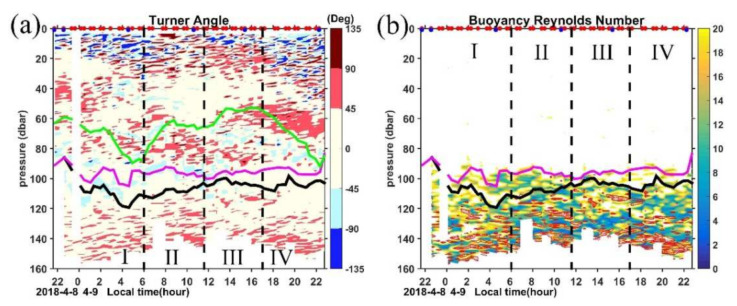
Time series of (**a**) Turner angle (Tu) and (**b**) buoyancy Reynolds number (R_eb_). In (**b**), the red contours mark the regions with 45° < Tu < 90°, only R_eb_ below the ML are color shaded, and above the ML, R_eb_ exceeding 20 are blanked. Purple and black lines mark the MLD and ILD, respectively. The green line in (**a**) represents the MLD calculated from the criteria of temperature difference of 0.3 °C according to Kara et al. (2003).

In addition to the high *ε* peaks induced by shear instability, there are also some high *ε* peaks where shear is weak during some MSP casts (Figure 9a,d). For example, during the cast conducted at 1820 LT, high *ε* values reaching 10^−8^∼10^−7.5^ W kg^−1^ exist around 120 m with the diapycnal diffusivity exceeding 10^−5^ m^2^ s^−1^, but the shears are relatively small, and the stratifications are moderate, so the *R_i_* cannot reach a critical value. Figure 12 shows the time series of vertical profiles of the vertical Turner angle (Tu) and the buoyancy Reynolds number (R_eb_). According to criteria listed in Table 1 in Lee et al. (2014) [38], we define three mixing regimes: turbulence is characterized as R_eb_ > 20 with −45° < Tu <45°, non-turbulent double diffusion is characterized as R_eb_ < 20 with −90° < Tu < −45° for diffusive convection or 45° < Tu < 90° for salt-fingering, and turbulent double diffusion is characterized as R_eb_ > 20 with −90° < Tu < −45° or 45° < Tu < 90°.

In the mixing layer above 40 m, the Tu shows a diurnal variation with patches of Tu < −90° dominating periods I and IV, suggesting the statically unstable conditions associated with enhanced buoyancy-driven mixing under nighttime convection (recall Figure 6a). Some patches of diffusive convection (−90° < Tu < −45°) can be identified in the top 15 m in these two periods, which are consistent with the condition of cold and fresh water above warm and salty water as indicated by the negative vertical temperature and salinity gradients in Figure 7b,d. During periods II and III, as the ocean is under heat gain, convection is reduced, and the Tu is mostly between 45° and 135°. Salt fingers with 45° < Tu < 90° dominate in the remnant layer where *ε* decays sharply from 10^−6.5^ W kg^−1^ in the top 15 m to 10^−8.5^ W kg^−1^ below 15 m in period III (Figure 6). Both the positive temperature and salinity gradients indicate a condition of warm and salty water above cold and fresh water and the low turbulence levels are favorable for active salt-fingering in this region.

Between 40 m and the base of ML, both salt finger and diffusive convection occur, with diffusive convection dominating. There is a patch of 45° < Tu < 90° and −90° < Tu < −45° between 50 dbar and 90 dbar in period IV. The temperature and salinity gradients in Figure 7b,d clearly show that there are two distinct temperature and salinity vertical gradients between 50–70 dbar and 70–90 dbar with positive (∂T/∂z> 0 and ∂S/∂z> 0) and negative (∂T/∂z< 0 and ∂S/∂z< 0) values occupying the upper and lower layers, respectively; however, these gradients are relatively small compared with those in the thermocline. The same sign of temperature and salinity gradients in this region suggests the opposite buoyancy effects of temperature and salinity on density.

**Figure 13:**
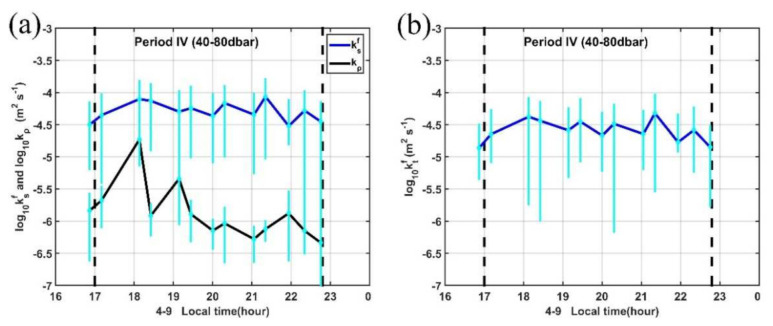
Vertical diffusivity of (**a**) salinity and (**b**) temperature due to salt fingering in the region between 40–80 dbar during period IV. The blue line is the averaged value with the 95% confidence level indicated by cyan lines. The black line in (**a**) is the diffusivities estimated from the observed dissipation rate.

Our MSP observation site is located in the western Pacific warm pool, where salinity stratification is known to be important. The large discrepancies between density and temperature-based mixed layer depth criteria may also suggest the existence of a density-compensated layer between the mixed layer and the interior [81,82,83]. The estimated mean salinity diffusivity by salt-fingering here is two to three orders of magnitude higher than that in other regions with the value ranging between 10^−4.5^∼10^−4^ m^2^ s^−1^ (Figure 13a). Values of temperature diffusivity by salt-fingering here is comparable but a little lower than that of salinity (Figure 13b), which is indicative of the effective salt transport in this regime. For Turner angles between 71.6° and 90° (2 > R_ρ_ > 1), double-diffusive salt fingers can induce enhanced diffusivity of both temperature and salt, corresponding to a high degree of density compensation of ∂T/∂z and ∂S/∂z [60]. The estimated diffusivities by salt-fingering are about one order of magnitude larger than those computed from the dissipation rate (Figure 13a), suggesting the contribution to vertical mixing by salt-fingering in this period. Thus, the relatively high R_eb_ (R_eb_ > 20, Figure 12b) and the moderate dissipation rate (10^−8.5^ W kg^−1^; Figure 6b) in this 50–90 dbar regime during period IV is indicative of a combination of turbulence and double-diffusive fingering.

In the BL, Tu is mostly between −45° and 45°, suggesting the doubly stable condition in this region. Patches of moderate *ε* in the BL in period I (Figure 6b) seem to be collocated with patches of double-diffusive convection (Figure 12a). Meanwhile, the relatively low R_eb_ during this period suggests the non-turbulent diffusive regime occurs in the BL. As to BL during periods III and IV, the large R_eb_ (>20) and doubly stable condition as indicated by Tu suggest classical three dimensional turbulence (Figure 12). When R_eb_ is less than 20, turbulence is suppressed by stratification and the buoyancy flux is suppressed [84].

Below the ML, where the TL is located, the deepening effect of vertical mixing and the stabilizing effect of stratification are in balance in the averaged sense [70]. As the stratification is increased compared with that in ML, turbulence is suppressed with R_eb_ < 20 at many depths (Figure 12b). Corresponding to those small R_eb_ patches, the Tu is mostly between −45° and 90°, suggesting either a doubly stable condition or salt-fingering condition in this region.

## 5. Discussion and Conclusions

Using direct oceanic microstructure measurements of velocity shear, temperature, and salinity in the upper 160 m, this study investigated characteristics of turbulence within the mixing layer, and the mixed layer and transition layer connecting to the upper thermocline during a continuous period of 25-h in the western Pacific NEC. The dynamics of diurnal turbulent mixing in the surface ML and upper thermocline in the NEC and the relationship between turbulent mixing and barrier layer evolution were examined. The major results of this study are as follows.

First, in the mixing layer, the order of magnitude values of *ε* are typically 10^−8^∼10^−7^ W kg^−1^ and exhibit an obvious diurnal cycle, whereas they drop to 10^−9^∼10^−8^ W kg^−1^ in the ML and upper thermocline. Below the ML, local shear instability induces some patches of high *ε* reaching 10^−8^∼10^−7.5^ W kg^−1^ (Figure S5).

Second, the BL is observed during 0200LT to 1000 LT in periods I and II, which is formed as a halocline between the upper fresh TSW and lower high salinity NPTW due to nighttime convection and wind gusts. Although the BL is also identified by the gradient criterion during 1400 LT to 2000 LT in periods III and IV, the relatively uniform salinity stratification and the large density ratio suggest that the salinity stratification in the BL during these two periods is weak. The significant decrease in salinity within the lower IL associated with the advection of relative fresh water by an anticyclonic eddy seems to contribute to the erosion of halocline during these two periods, and the gradient criterion may not be applicable in identifying the BL under this situation.

Third, by combing N^2^ and *S*^2^, variations in *ε* in the TL can be predicted by the scaling $\varepsilon ∼{\left|S\right|}^{-0.40}{\left|N\right|}^{0.20}$. This simple scaling of turbulent dissipation rates in the TL relevant to shear instability and stratification provides a way for future efforts to improve the vertical mixing in upper western Pacific NEC in coarse resolution models.

As advection associated with the AE and doubly diffusive phenomena are both active in the upper WPNEC during the observation, the diapycnal diffusivity estimation based on Osborn’s (1980) [48] model may not applicable here due to its exclusion of these two processes. Meanwhile, the fixed value used in the gradient criterion for BL identification may not be appropriate under the influence of anticyclonic eddies, when the ML and IL are both deepened. In eddy-rich regions, the formation of a BL could be largely influenced by these eddies, which may be ignored in its climatology field based on heavily smoothed climatological fields of hydrographic data using the gradient criterion.

In addition to the high *ε* peaks induced by shear instability, double-diffusive mixing also contributes to the diurnal variations of turbulent and vertical mixing levels in the upper WPNEC. These findings reported in this study contribute to advancing our understanding of the role played by upper-ocean layer turbulence in the water mass transformation and air–sea interaction of the NEC in the western Pacific Ocean.

The main findings of the present study are the observed BL associated with the NPTW in spring and the significant influence of the advection of a mesoscale eddy on the BL during a 25-h period. We also provide a scaling relationship between the dissipation rate and shear instability and stratification in the transition layer. Given that these results are based on 25-h MSP measurements in April, this can only serve as one sample for the diurnal cycle. The dominant dynamics of diurnal turbulent mixing in the surface ML and upper thermocline in the NEC differ depending on the sea state, more or less altered/turbulent (strong or light winds, breaking waves, storms, etc.), and such conditions manifest themselves more or less at different times of the year. Therefore, a sufficiently representative picture requires data recorded at different times of the year in future observations.

## Figures and Tables

**Table 1:** Mean values of wind speed (${\bar{U}}_{10}$ ), surface buoyancy flux ($\bar{J_{B}^{0}}$ ), Monin–Obukhov length ($\bar{L}$ ), mixed layer depth ($\bar{D}$ ), barrier layer thickness ($\bar{B}$ ), surface heat flux ($\bar{J_{q}^{0}}$ ), and number of MSP casts (N).

	${\bar{U}}_{10}$	$\bar{J_{B}^{0}}$	$\bar{L}$	$\bar{D}$	$\bar{B}$	$\bar{J_{q}^{0}}$	**N**
	**(m/s)**	**(W kg^−1^)**	**(m)**	**(m)**	**(m)**	**(W m^−2^)**	
Period I	7.84	−2.40 × 10^−7^	−33.19	96.71	11.19	−515.89	18
Period II	9.34	1.42 × 10^−7^	27.22	95.97	12.27	304.71	11
Period III	9.29	1.39 × 10^−7^	28.29	96.53	6.58	297.97	12
Period IV	8.84	−3.32 × 10^−7^	−20.39	94.08	9.67	−741.04	12

## Data Availability

Daily gridded maps of merged absolute dynamic topography heights (MADT-H) and absolute geostrophic velocities (MADT-UV) from Aviso+ (http://www.aviso.altimetry.fr, accessed on 26 January 2022) and Copernicus Marine Environment Monitoring Service (CMEMS http://marine.copernicus.eu/, accessed on 26 January 2022) are used to infer the surface currents during the period of the MSP observations. The 10-m wind stress vector, air temperature, and pressure were measured by the automatic weather station installed on the R/V Xiangyanghong 18. Longwave radiation and shortwave radiation data computed by the European Centre for Medium-Range Weather Forecasts (ECMWF) forecast system with a resolution of 0.125° × 0.125° were used in the buoyancy flux calculation (Berrisford et al. 2011; http://apps.ecmwf.int/datasets/, accessed on 26 January 2022).

## Supplementary Materials

The following are available online at https://www.mdpi.com/article/10.3390/s22031167/s1. Figure S1. The sinking velocity of the profiler during the 25-h observation. Figure S2. The geostrophic velocity anomalies (arrows) on (a) 8 April, (b) 9 April, and (c) 10 April in 2018 from altimetry data with the synchronous sea surface height anomalies (SSHA) being superimposed. The purple diamond shows the microstructure profile measurement station (142.15° E, 11.25° N). Figure S3. Evolution of (a) the ILD and MLD, (b) SST, and (c) SSS during period IV. Figure S4. Profiles of n^2^ with density inversion occurring during the four periods. Figure S5. The scatter plot of stratification versus dissipation rate in the mixing layer (green), the mixed layer (gray), the transition layer (black), and below the mixed layer (blue).

## Abbreviations

*ε*	Turbulent Kinetic Energy Dissipation Rate
WPNEC	Western Pacific North Equatorial Current
ML	Mixed Layer
NEC	North Equatorial Current
KC	Kuroshio Current
MC	Mindanao Current
NPTW	North Pacific Tropical Water
BL	Barrier Layers
IL	Isothermal Layer
TL	Transition Layer
MSP	Microstructure Profile
LT	Local Time
LADCP	Lowered Acoustic Doppler Current Profiler
CTD	Conductivity, Temperature, Depth
AE	Anticyclonic Eddy
TSW	Tropical Surface Water
ITCZ	Intertropical Convergence Zone
