# Supplementary material for: Direct Measurements of Turbulence in the Upper Western Pacific North Equatorial Current over a 25-h Period
Source: Sensors (Basel). 2022 Feb 3;22(3):1167. doi: 10.3390/s22031167 (PMC8839716; doi:10.3390/s22031167)
Supplement: Supplementary file 1 [file sensors-22-01167-s001.zip › sensors-1545565-supplementary.pdf]

Supplementary Materials: The following are available online at www.mdpi.com/xxx/s1. Figure S1. The sinking velocity of the profiler during the 25-h observation. Figure S2. The geostrophic velocity anomalies (arrows) on (a) 08 April, (b) 09 April, and (c) 10 April in 2018 from altimetry data with the synchronous sea surface height anomalies (SSHA) being superimposed. The purple diamond shows the microstructure profile measurement station (142.15° E, 11.25° N). Figure S3. Evolution of (a) the ILD and MLD, (b) SST, and (c) SSS during period IV. Figure S4. Profiles of n^2 with density inversion occurring during the four periods. Figure S5. The scatter plot of stratification versus dissipation rate in the mixing layer (green), the mixed layer (gray), the transition layer (black), and below the mixed layer (blue).

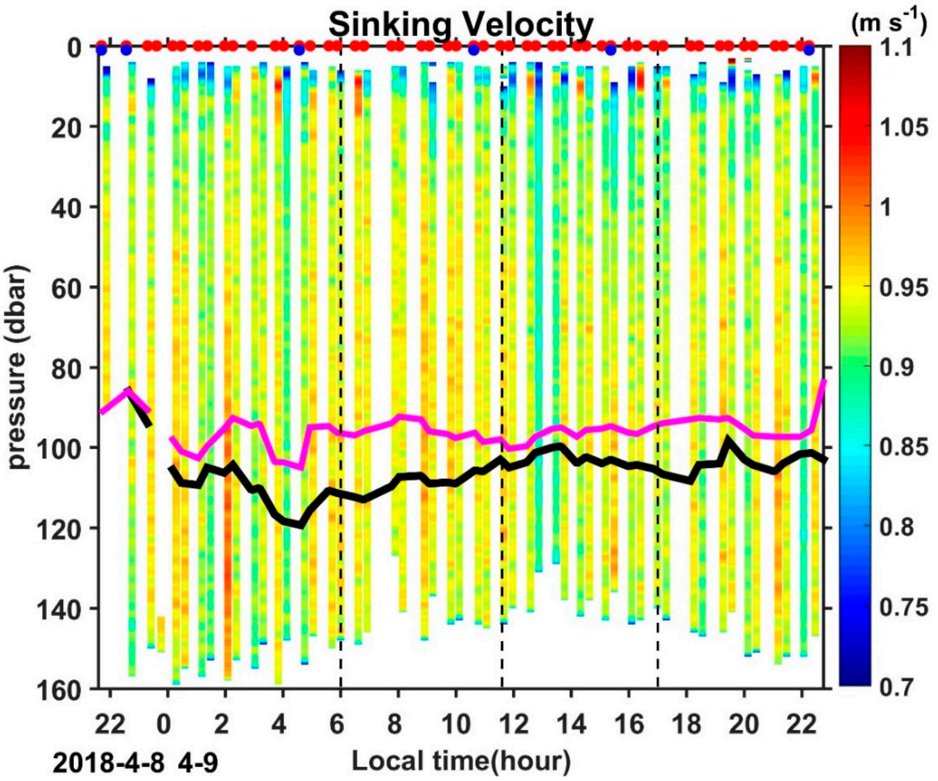

Figure s1. The sinking velocity of the profiler during the 25-h observation.

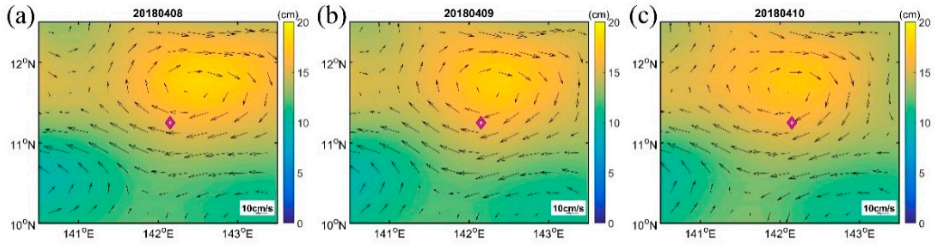

Figure s2. The geostrophic velocity anomalies (arrows) on (a) Apr. 08, (b) Apr. 09 and (c) Apr. 10 in 2018 from altimetry data with the synchronous sea surface height anomalies (SSHA) being superimposed. The purple diamond shows the microstructure profile measurement station (142.15°E, 11.25°N).

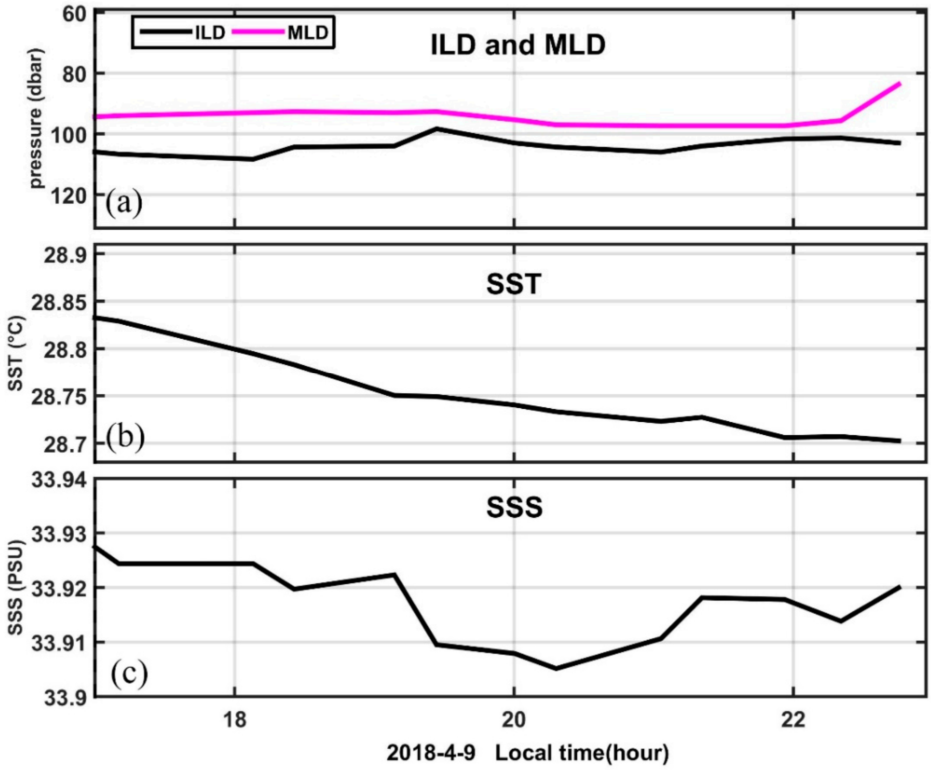

Figure s3. Evolution of (a) the ILD and MLD, (b) SST, and (c) SSS during period IV.

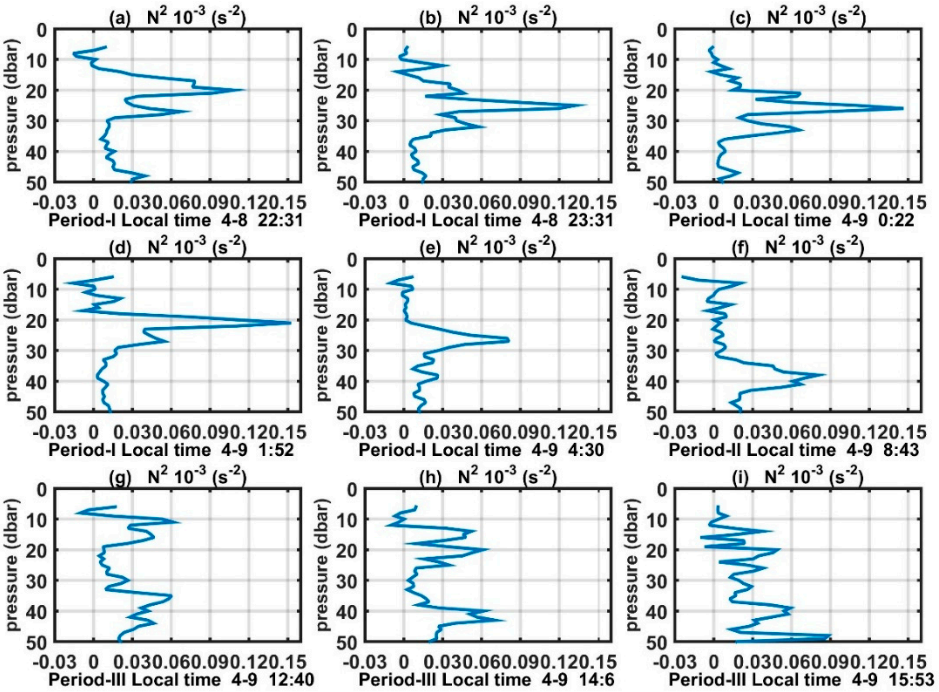

Figure s4. Profiles of N^2 with density inversion occurring during the four periods.

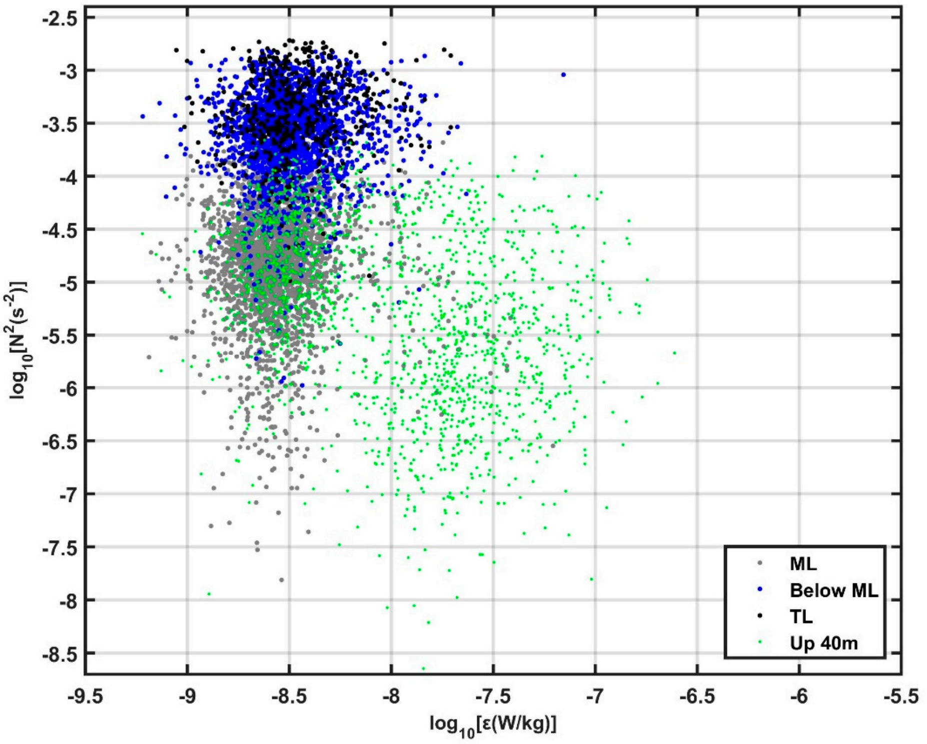

Figure s5. The scatter plot of stratification versus dissipation rate in the mixing layer (green), the mixed layer (gray), the transition layer (black), and below the mixed layer (blue).
